# Attenuated activation of the unfolded protein response following exercise in skeletal muscle of older adults

**DOI:** 10.18632/aging.102273

**Published:** 2019-09-14

**Authors:** Corey R. Hart, Zachary C. Ryan, Kyle T. Pfaffenbach, Surendra Dasari, Mojtaba Parvizi, Antigoni Z. Lalia, Ian R. Lanza

**Affiliations:** 1 Division of Endocrinology and Metabolism, Mayo Clinic College of Medicine, Rochester, MN 55905, USA; 2 Division of Biostatistics and Informatics, Mayo Clinic College of Medicine, Rochester, MN 55905, USA; 3 Department of Physical Activity and Health, Eastern Oregon University, La Grande, OR 97850, USA

**Keywords:** aging, skeletal muscle, exercise, unfolded protein response, ER stress

## Abstract

Sarcopenia is linked with impaired adaptive responses to exercise in aging skeletal muscle. The unfolded protein response (UPR) is an important intramyocellular molecular response pathway that is activated by exercise. The influence of age on skeletal muscle adaptive UPR in response to exercise, and the relationship to other key exercise-responsive regulatory pathways is not well-understood. We evaluated age-related changes in transcriptional markers of UPR activation following a single bout of resistance exercise in 12 young (27 ± 5yrs) and 12 older (75 ± 5yrs) healthy men and women. At baseline, there were modest differences in expression of UPR-related genes in young and older adults. Following exercise, transcriptional markers of UPR pathway activation were attenuated in older adults compared to young based on specific salient UPR-related genes and gene set enrichment analysis. The coordination of post-exercise transcriptional patterns between the UPR pathway, p53/p21 axis of autophagy, and satellite cell differentiation were less evident in older compared to young adults. In conclusion, transcriptomic analysis revealed an age-related decline in the adaptive UPR transcriptional response following a single bout of exercise that could contribute to impaired exercise responsiveness with age.

## INTRODUCTION

Aging is associated with the loss of skeletal muscle mass, quality, and function; decrements that have a negative influence on health span [1]. When skeletal muscle function deteriorates to the point that activities of daily living can no longer be performed independently, quality of life is compromised, and risk of disability, morbidity, and mortality increases substantially [1]. Resistance exercise improves muscle mass and function, but there is emerging evidence that the molecular and cellular responses to anabolic stimuli (e.g., exercise and nutrition) are attenuated in older adults; a phenomenon termed anabolic resistance [2]. In the quest to understand the molecular drivers of age-related anabolic resistance, the physiological unfolded protein response (UPR) has emerged as a key regulatory pathway in skeletal muscle protein quality control and adaptations to exercise [3–6]. Early evidence points to altered UPR as an explanation for age and disease related changes in protein folding and accumulation and aggregation of proteins within the endoplasmic reticulum (ER) [7–10].

The UPR is a highly conserved cellular stress response within the ER. Accumulation of misfolded proteins in the ER lumen trigger ER stress and subsequent initiation of the UPR, which is mediated by three ER transmembrane sensors: RNA-dependent protein kinase-like ER eukaryotic translation initiation factor 2 alpha kinase (PERK), inositol-requiring protein 1 (IRE1), and activating transcription factor-6 (ATF6) [11]. Detection of misfolded or unfolded proteins by the ER triggers the dissociation of glucose-regulated protein 78 (Grp78) from PERK, IRE1, and ATF6, initiating a signaling cascade to restore proteostasis within the cell [12]. PERK phosphorylates eukaryotic translation initiation factor 2α (eIF2α) to attenuate mRNA translation [11, 13]. IRE1 phosphorylation promotes the activation and splicing of X-box-binding protein 1 (XBP1s) mRNA [11, 14]. ATF6 translocates to the Golgi, where it is processed by proteases and releases the active cytosolic fragment (ATF6f), which controls genes responsible for encoding ER associated degradation (ERAD) and XBP1 [11]. Taken together, the UPR signaling cascade modulates chaperone and protein expression under physiological conditions to maintain proteostasis and prevent future ER stress [15].

The role of the UPR has been studied and characterized in several physiological and pathologic conditions. Components of the UPR play a role in feeding [16], exercise adaptations [3], and chronic diseases including diabetes, obesity, Alzheimer’s, and cancer [17] in tissues such as in the liver [18], pancreas [19], and adipose tissue [20]. Under resting conditions, tonic activation of the UPR in skeletal muscle has been implicated in mechanisms that both prevent [21] and contribute [22, 23] to age-associated sarcopenia and anabolic resistance. Selective activation of UPR branches, sometimes referred to as the adaptive UPR, in response to both aerobic [3] and resistance exercise [5] demonstrate an important role of the UPR in mediating skeletal muscle adaptations. However, less studied is the extent to which age influences markers of the adaptive UPR in skeletal muscle in response to exercise, and the relationship to other key exercise-responsive regulatory pathways (e.g., autophagy, hypertrophy, mitochondrial biogenesis). We recently observed that healthy older adults exhibited decreased muscle strength accompanied by reduced muscle mitochondrial function and attenuated transcriptional responses to a single bout of exercise compared to young adults [24]. Here we leveraged the availability of residual biospecimens to evaluate age-related alterations in markers associated with UPR pathway activation in response to a single bout of resistance exercise in young and older adults.

## RESULTS

### Post-exercise UPR pathway activation attenuated with age

The characteristics of the twelve young and older men and women have been previously described [24] and are provided in in Table 1. Most notably, there were no significant differences between the young and older adults in BMI, total body fat percentage, total skeletal muscle index (SMI), or fat and lean mass in the legs. SMI is a morphological calculation used to define sarcopenia [25, 26]. While there was no significant difference in absolute VO_2_ peak (L/min) between young and older adults, VO_2_ peak normalized to body mass, absolute leg strength (1RM), and leg strength relative to fat free mass in the leg were significantly (p≤0.05) decreased in older compared to young adults.

**Table 1:** Subject Characteristics.

	**Young N=12**	**Older N=12**		**P Age**
**Physical characteristics**				
Age (years)	27 ± 5	76 ± 5		
Sex (F/M)	6F/6M	7F/5M		
Height (cm)	169.6 ± 3.7	163.7 ± 8.5		0.03*
Weight (kg)	69.9 ± 7.0	70.7 ± 12		0.08
BMI (kg/m^2^)	24.3 ± 2.7	26.3 ± 2.8		0.10
SBP (mmHg)	112.4 ± 11.8	124.2 ± 11.6		0.02*
DBP (mmHg)	69.9 ± 9.7	64.3 ± 10.5		0.18
**Body composition**				
Total body Fat (%)	31.7 ± 7.7	36.6 ± 5.9		0.10
Fat, arms (kg)	2.0 ± 0.5	2.5 ± 0.7		0.04*
Fat, legs (kg)	7.6 ± 2.2	7.8 ± 2.2		0.82
Fat, trunk (kg)	10.6 ± 2.7	13.7 ± 5.1		0.08
Total lean mass (kg)	45.5 ± 7.8	42.5 ± 7.3		0.30
Lean, arms (kg)	5.0 ± 1.2	4.5 ± 1.1		0.31
Lean, legs (kg)	15.6 ± 3.1	13.9 ± 2.5		0.15
Lean, trunk (kg)	22 ± 3.6	21.1 ± 3.6		0.51
**Physical function**				
SMI (kg/m^2^)	15.8 ± 2.6	15.8 ± 1.4		0.67
1RM (AU)	84.4 ± 21.4	52.1 ± 14.6		0.001*
1RM (AU/kg FFM)	1.74 ± 0.2	1.15 ± 0.19		<0.0001*
VO_2_ peak (L/min)	2.2 ± 0.5	1.5 ± 0.4		0.10
VO_2_ peak (ml/kg/min)	31.4 ± 5.6	21.7 ± 4.2		<0.001*

M; Male, F; Female, BMI; body mass index, SBP; systolic blood pressure, DBP; diastolic blood pressure, SMI; total skeletal muscle index, 1RM; 1 repetition maximum, AU; arbitrary units. VO_2_ peak; peak oxygen uptake. Data are shown as mean ± SD, * p ≤ 0.05, Wilcoxon rank sum test for age group comparisons and for non-parametric distributions.

**Figure 1:**
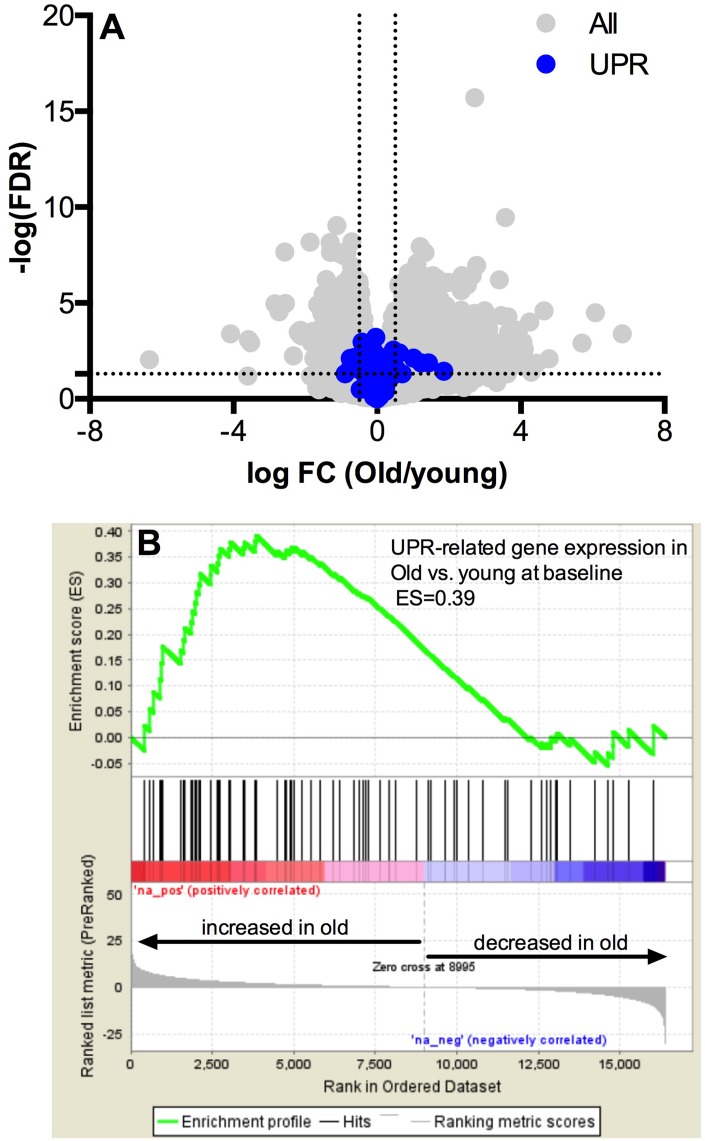
**Pathway analysis of the unfolded protein response mRNA transcripts at baseline.** Baseline UPR genes transcripts are not different at baseline in young vs. older adults (**A**) The x-axis represents the log fold change, while y-axis represents the –log FDR-value for each gene. UPR genes are colored in blue. Dotted vertical lines represent the threshold for statistical significance (FDR < 0.05, −log [FDR value] > 1.303). Gene set enrichment analysis (GSEA) of 72 gene transcripts associated with the UPR reveals a trend towards upregulation of mRNA for the unfolded protein response in skeletal muscle under basal conditions (**B**) in the older compared to younger adults (FDR < 0.05 and an absolute log^2^ fold change R ≥ 0.5).

**Figure 2:**
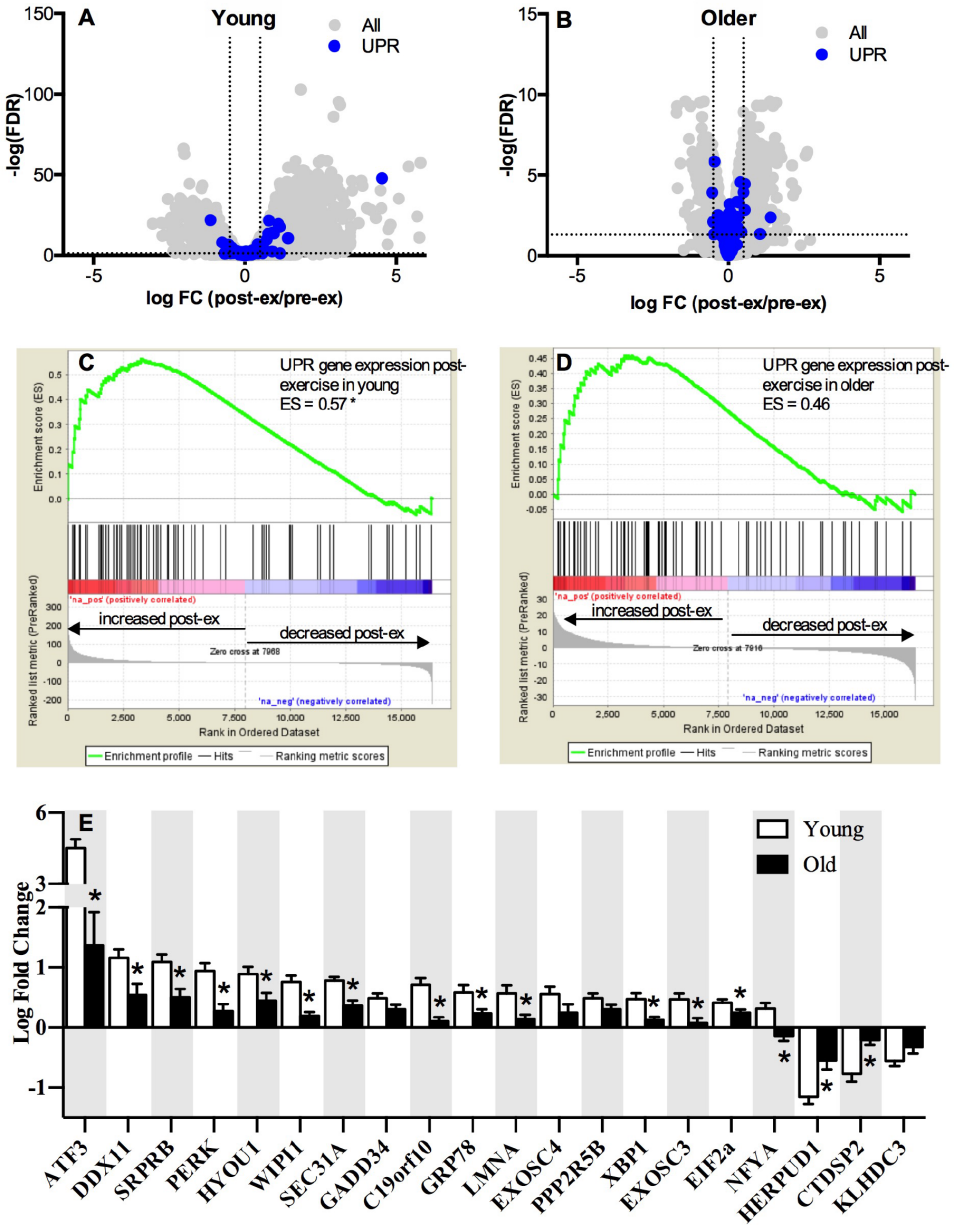
**Pathway analysis of the unfolded protein response mRNA transcripts post-exercise.** The unfolded protein response (UPR) in skeletal muscle volcano plots of mRNA expressed as a log fold change from baseline to 18h post-exercise in young (**A**) and older (**B**) adults. The x-axis represents the log fold change, while y-axis represents the –log FDR-value for each gene. Dotted vertical lines represent the threshold for statistical significance (FDR < 0.05, −log [FDR value] > 1.303). Gene set enrichment analysis (GSEA) was performed using Broad’s GSEA software for 72 genes related to UPR pathway and revealed a significantly greater upregulation of the UPR pathway between rest and 18h post-exercise in young (**C**) compared to older (**D**) adults (FDR < 0.05 and an absolute log2 fold change ≥ 0.5). (**E**) UPR up- and down-regulated mRNAs significantly altered (FDR < 0.05 and an absolute fold change of ≥ 1.2) between baseline and 18h post-exercise in young and older adults, sorted by the log2 fold change. *NS* = not significantly altered.

**Figure 3:**
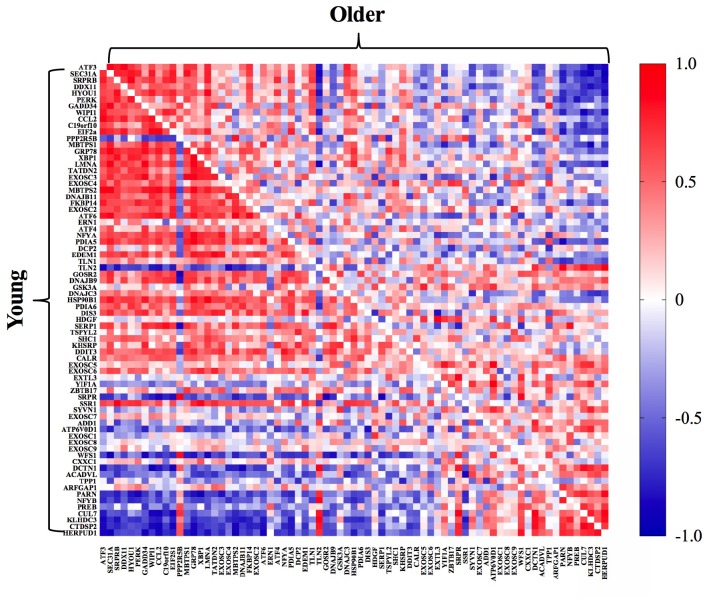
**Pathway activation analysis demonstrated a stronger coordinated response of gene transcripts associated with the UPR post-exercise in young (bottom left) compared to older (upper right) adults.** The correlation matrix heat maps demonstrate significant positive (dark red) and negative (dark blue) relationships between the fold-change from baseline to 18h post-exercise for the individual gene transcripts associated with the UPR pathway in young and older adults.

The whole-muscle transcriptome dataset generated from RNA sequencing was used to identify age-related differences in the UPR pathway using gene set enrichment analysis (GSEA). The GSEA calculates an enrichment score (ES), which represents how frequently genes of the UPR pathway occur at the top or bottom of the overall ranked dataset [27], thereby providing a readout of the coordination of multiple genes within the UPR pathway. At baseline before exercise, transcripts in the UPR pathway were not remarkably different between young and older adults (Figure 1A) and the overall enrichment of the UPR pathway visualized by GSEA was not significantly enriched in older adults, although there was a notable trend (ES = 0.39, FDR = 0.0842) toward increased tonic UPR activation in older adults (Figure 1B). Age-related differences in UPR activation following exercise were assessed from the fold change (pre- to post-exercise) in the UPR-associated gene transcripts (Figure 2). The volcano plots in Figure 2 demonstrate that UPR-related genes are influenced by an acute exercise bout to a greater extent in young (Figure 2A) compared to older adults (Figure 2B). Subsequent GSEA further quantified these age-associated differences and revealed a significant increase in UPR pathway activation in young (ES = 0.57, FDR = 0.0405, Figure 2C) that was not statistically significant in older adults (ES = 0.46, FDR = 0.0619, Figure 2D). Examination of individual salient UPR-related genes reveals attenuated changes post-exercise in older adults compared to young (Figure 2E). The overall coordinated response to exercise among all detected UPR-related genes was stronger in young (Figure 3) compared to older adults (Figure 3), as evidenced by stronger correlations among UPR pathway gene transcripts from baseline to 18 hours post-exercise. There were no age-related differences in the expression of proteins involved in the UPR from baseline to 18 hours post-exercise (Supplementary Figure 2). Expression of phosphorylating proteins that are indicative of ER stress and UPR pathway activation typically peak at 48 hours post-exercise [4, 5], which could explain why no differences were detected in the present study.

Although the small sample size of this study precludes an adequately powered assessment of sex differences or age-by-sex interactions in the post-exercise UPR transcriptional response using GSEA, a preliminary comparison of individual UPR gene transcripts in males and females within each age group suggests that sex differences may exist. For example, in the young adults the pre-to-post log change in PERK was significantly (p≤0.05) greater in females (1.24 ± 0.19) than males (0.64 ± 0.41), while in older adults the pre-to-post log change was significantly (p≤0.05) greater in the females than males for ATF3 (females: 2.54 ± 1.76, males: 0.19 ± 1.36) and Grp78 (females: 0.40 ± 0.21, males: 0.07 ± 0.25). When age groups were combined to examine sex effects irrespective of age, the pre-to-post log changes in key UPR gene transcripts were significantly (p≤0.05) greater in females compared to males (ATF3: females: 3.71 ± 1.74, males: 1.36 ± 1.94; PERK: females: 0.84 ± 0.50, males: 0.27 ± 0.40; Grp78: females: 0.61 ± 0.38, males: 0.24 ± 0.24; and XBP1: females: 0.40 ± 0.36, males: 0.12 ± .18).

### The PERK arm of the UPR pathway and the relationship to autophagy and satellite cell gene transcripts

**Figure 4:**
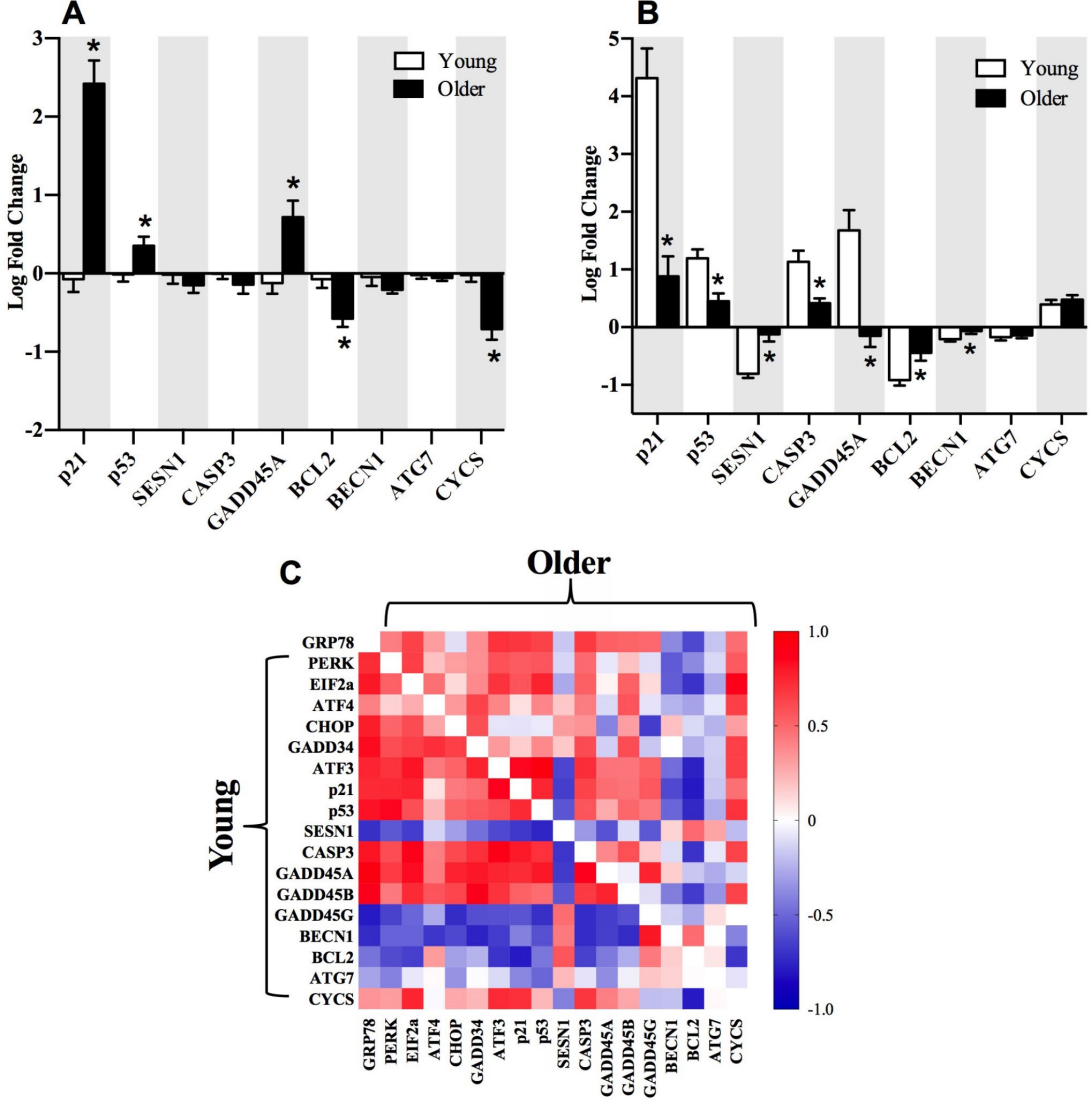
**Selected gene transcripts associated with p53/p21 axis of autophagy and the relationship to the PERK arm of the UPR.** Gene transcripts associated with p53/p21 axis are elevated at baseline (**A**) and decreased 18h post-exercise (**B**) in older compared to younger adults. Correlations between gene transcripts associated with the PERK arm of the UPR and p53/p21 axis post-exercise (**C**) in young (bottom left) compared to older (upper right) adults. The correlation matrix heat maps demonstrate significant positive (dark red) and negative (dark blue) relationships between the fold-change from baseline to 18h post-exercise for the individual gene transcripts associated with the UPR pathway in young and older adults. *Significantly (p ≤ 0.05) different between groups. Data bars are presented as mean ± SEM.

When we interrogated UPR-related transcripts specific to each arm of the UPR, we observed that genes associated with the PERK arm were most enriched in young adults post-exercise (Figure 2E). Specifically, PERK and ATF3 mRNA exhibited a significantly greater fold change from baseline to 18 hours post-exercise in young compared to older adults. Emerging evidence supports crosstalk between the UPR and autophagy to restore cellular homeostasis [28]. In the present study, the gene transcripts associated with the p53/p21 axis of autophagy were significantly elevated in older compared to young adults at baseline (Figure 4A), but the fold change 18 hours post-exercise in these genes was attenuated in older adults (Figure 4B). As observed with the overall UPR pathway activation, young adults exhibited a more coordinated response between genes associated with the PERK arm of the UPR and the p53/p21 axis, indicated by the associations between fold change in salient gene transcripts from baseline to 18 hours post-exercise in young compared to older adults (Figure 4C and Supplementary Figure 3). Similar to UPR-related transcripts, preliminary sex differences were evident for p53/p21 gene transcripts. Young females exhibited significantly (p≤0.05) greater pre-to-post exercise log fold change in p21 (females: 5.18 ± 0.62, males: 2.88 ± 2.47), p53 (females: 1.54 ± 0.41, males: 0.74 ± 0.51), CASP3 (females: 1.56 ± 0.32, males: 0.64 ± 0.91), and GADD45A (females: 2.47 ± 1.29, males: 0.65 ± 1.31) compared to young males, and BECN1 was significantly (p≤0.05) downregulated in young females (-0.33 ± 0.10) compared to young males (-0.10 ± 0.14). When age groups were combined to examine sex effects irrespective of age, the pre-to-post log changes were significantly (p≤0.05) greater in females compared to males for p21 (females: 3.71 ± 1.74, males: 1.36 ± 1.94), p53 (females: 1.12 ± 0.52, males: 0.54 ± 0.64), SESN1 (females: 0.96 ± 0.45, males: 0.50 ± 0.47), CASP3 (females: 0.84 ± 0.50, males: 0.27 ± 0.40), GADD45A (females: 0.85 ± 0.45, males: 0.45 ± 0.45), BCL2 (females: 0.51 ± 0.34, males: 0.19 ± 0.23), BECN1 (females: 0.69 ± 0.30, males: 0.36 ± 0.29), and CYCS (females: 0.42 ± 0.41, males: 0.10 ± 0.23). Despite some intriguing evidence supporting potential sex differences in post-exercise UPR and autophagy-related transcripts, these trends should be interpreted cautiously due to the fact that this study was designed to be inclusive of both sexes, but was not powered to support a formal interrogation of sex differences or age-by-sex interactions.

**Figure 5:**
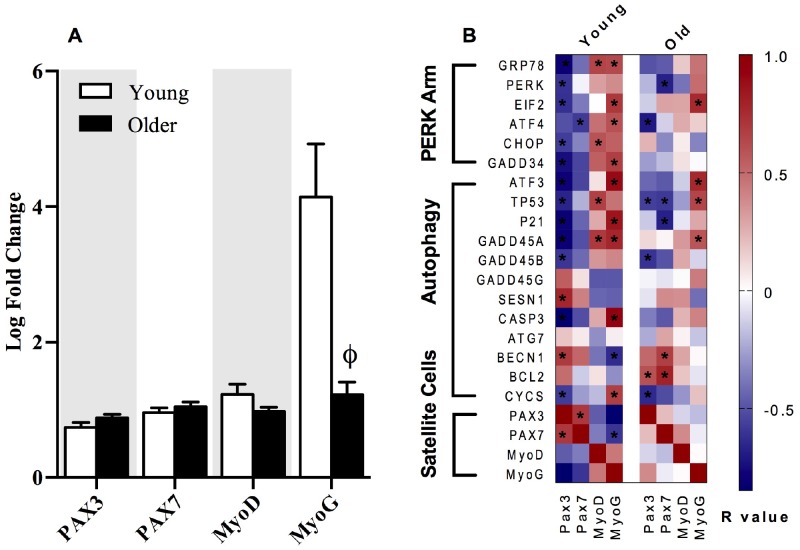
**The relationships between markers of satellite cell differentiation, the p53/p21 axis of autophagy and the PERK arm of the UPR.** The fold change from baseline to 18h post-exercise in gene transcripts representing satellite cell differentiation in young and older adults (**A**). Correlations demonstrate significant positive (dark red) and negative (dark blue) relationships between the fold-change from baseline to 18h post-exercise for the individual gene transcripts associated with the satellite cells differentiation markers (Pax3, Pax7, MyoD, MyoG), the p53/p21 axis, and the PERK arm of the UPR pathway in young and older adults (**B**). Data bars are presented as mean ± SEM. *Significant correlation (p ≤ 0.05). † Significantly different (p ≤ 0.05) between young and older adults.

Both p21 and PERK have recently been linked to alterations in satellite cell homeostasis and regenerative myogenesis in skeletal muscle [6]. When the metabolic demand on skeletal muscle is low, satellite cells reside in a quiescent state and are activated during periods of disrupted homeostatic imbalance to produce myonuclei, induce hypertrophy, and repair myofibers for muscle regeneration [29]. Skeletal muscle satellite cells express the paired box (PAX) transcription factors Pax3 and Pax7. Satellite cells express high levels of Pax7 in the quiescent state, while Pax7 expression declines in satellite cells upon differentiation during muscle regeneration and repair [30]. Conversely, Pax3 has been documented to be expressed in satellite cells during both the quiescent and activated state [30]. In the present study, transcripts corresponding to satellite cell markers, Pax3, Pax7, and MyoD were similar in young and older adults at baseline and 18 hours post-exercise, but there was a clear attenuation of MyoG expression post-exercise in older adults (Figure 5A). Furthermore, gene transcripts associated with the PERK arm of the UPR and p53/p21 axis exhibited stronger correlations with Pax3, Pax7, MyoD, and MyoG in young compared to older adults when expressed as a fold change from baseline to 18 hours post-exercise (Figure 5B).

## DISCUSSION

Stress response pathways are critical to maintaining healthspan, yet many of these pathways are impaired with aging [31] in conjunction with skeletal muscle impairments commonly observed after the seventh decade of life [32]. The present study was designed to interrogate the transcriptional response of the UPR pathway to a single bout of resistance exercise in skeletal muscle of healthy young and older adults. We found that the post-exercise changes in UPR-associated gene transcripts were attenuated in older adults. Furthermore, the coordination of post-exercise gene expression patterns between the UPR pathway, p53/p21 axis of autophagy, and satellite cell differentiation were less evident in older compared to young adults. These data suggest that the transcriptional UPR pathway response to resistance exercise is attenuated with age, even in the absence of frailty or chronic disease. Further, the lower UPR response to resistance exercise observed with aging coincides with a decline in other key regulatory pathways known to be important in maintenance of skeletal muscle function [1].

Few studies have assessed ER stress and activation of the UPR following an acute bout of exercise in skeletal muscle [3–5, 33, 34] and far fewer have evaluated aging as a modifier of post-exercise UPR activation in skeletal muscle [4, 5]. Our data support the possibility that the transcriptional patterns of skeletal muscle UPR activation following exercise are attenuated in healthy older adults; a conclusion that contrasts two previous studies where young and older males demonstrated similar post-exercise mRNA fold changes of Grp78, IRE1, PERK, ATF6, ATF4, CHOP, eIF2α, and GADD34, and protein expression of Grp78, IRE1, and PERK [4, 5]. Evidence of age and sex influences on skeletal muscle adaptations to resistance exercise has been previously documented [35]. Despite some initial evidence of sex-related differences in the log fold change of individual UPR and autophagy gene transcripts post-exercise between females and males in the present study, we did not feel confident in our ability to evaluate any sex by age interaction with sufficient statistical power due to the relatively low number of females within each age group. Future studies are needed to evaluate potential sex-related differences in UPR pathway activation following exercise with advancing age.

Skeletal muscle adaptations to exercise are mediated by a myriad of metabolic and molecular pathways [36] and the coordination among these pathways identifies patterns of coexpression that assist in the identification of potential molecular signals contributing to skeletal muscle function in the context of aging and disease. A progressive decline in the adaptive UPR, characterized by a decline ER molecular chaperones and protein folding capacity, has been implicated in the age-associated reduction of cellular functions in the brain, liver, pancreas, and spleen [37, 38]. Prior evidence suggests an early link between chronic UPR activation and anabolic resistance in skeletal muscle through the inhibition of mTORC1 [22, 23]. However, in the present study, age-related differences in the magnitude and coordination of a large number of UPR-associated gene transcripts post-exercise were present in the absence of any significant differences in the UPR pathway activation at baseline between young and older adults (Figure 1), suggesting that chronic UPR activation did not influence the age-related differences in markers of UPR pathway activation post-exercise. Alternatively, chronic low-grade inflammation and oxidative stress appear to influence the pattern of downstream UPR pathway activation in pancreatic beta cells [39]. It is therefore interesting to note that systemic concentrations of interleukin-6 and tumor necrosis factor alpha were elevated in older adults in this study as reported previously [24]. Based on these observations, the influence of chronic, low-grade systemic inflammation on the adaptive potential of the UPR in skeletal muscle post-exercise merits further investigation.

In the present study, correlations between the fold change of transcripts associated with the PERK arm of the UPR and the p53/p21 axis of autophagy post-exercise were stronger in young (Figure 4C) compared to older adults (Figure 4D). Unaccustomed exercise can evoke damage to cellular components which can be degraded in lysosomes through the process of autophagy [40]. Prior evidence indicates that ER stress induces autophagy in skeletal muscle [41]. The tumor suppressor protein, p53, is responsible for cell repair and enhancing cell survival in response to stress [42]. Acute activation of p53 attenuates the functional decline in skeletal muscle with age through another tumor suppressor protein, p21 [43]. Recent studies show acute induction of autophagy in skeletal muscle 48 hours following resistance exercise, as well as an age-related decline in this adaptive stress response [4]. However, increased p53 and p21 expression under resting conditions has been associated with senescence and an aging phenotype in skeletal muscle [44]. Indeed, in the present study, older adults exhibited elevated p53 and p21 mRNA expression at baseline (Figure 4A), but the post-exercise fold change in gene transcripts associated with the p53/p21 axis of autophagy was blunted in older compared to younger adults (Figure 4B). In support of an age-related difference in post-exercise autophagy activation in skeletal muscle, Hentilä and colleagues [4] recently reported an increase in lapidated LC3II, an indicator of autophagosome content, 48 hours following a single bout of unaccustomed resistance exercise, which persisted after 21 weeks of resistance training in young, but not in older men. Further studies are needed to evaluate the impact of age on the relationship between autophagy machinery induced by resistance exercise and UPR activation.

Satellite cells (SC) have been documented to serve an important role in the adaptation of skeletal muscle to resistance training [45, 46]. Upon mechanical loading or injury, SC are activated, proliferate, further differentiate, and fuse onto regenerating muscle fibers [47]. Activation of SC is coordinated by the up- or down-regulation of paired box transcription factors 3 and 7 (Pax3 and Pax7) and the myogenic regulatory factors myogenin 5, D, and G (Myf5, MyoD, and MyoG respectively) [48]. The sequential myogenic program of SC markers are characterized in the quiescent state as Pax7+/Myf5-/MyoD-/MyoG-, followed by Pax7+/Myf5+/MyoD-/MyoG- in the proliferative state, and Pax7-/Myf5-/MyoD-/MyoG+ in the differentiated state before returning to the quiescent state upon muscle repair or regeneration [48]. A reduced return to quiescence accompanied by a decrease in the SC pool size with advancing age has been attributed to reduced myonuclear accretion and hypertrophy in skeletal muscle in response to exercise [48, 49]. In the present study, the fold change in post-exercise MyoG was significantly decreased in older compared to young adults, although there were no age-related differences in Pax3, Pax7, or MyoD mRNA (Figure 5A). A coordinated response between gene transcripts associated with SC, autophagy, and the PERK arm of the UPR was more apparent in young compared to older adults (Figure 5B). The significant inverse correlations between both the PERK arm of the UPR and autophagy in relationship to SC gene transcripts suggests a potential role in myogenic transitioning of SC from the quiescent to the fully differentiated state. Indeed, recent evidence supports a pivotal role of the PERK arm in the regulation of SC homeostasis during regenerative myogenesis following injury [6] and for the preservation of skeletal muscle mass and function [7], although the underlying mechanism by which the PERK arm regulates hypertrophy and expansion of the SC pool size in response to exercise is unclear at this time.

We recognize some notable limitations to the present study. UPR activation was assessed 18 hours after acute exercise, which represents an intermediate time point compared to prior studies which evaluated UPR activation at 1-3 hours or 24–48 hours post-exercise [4, 5]. We recognize our ability to definitively link the measured blunted adaptive UPR transcriptional response with robust changes phosphorylation of key UPR and p53/p21 autophagy proteins in older adults was hindered by non-optimal timing of muscle biopsies. Indeed, prior evidence suggests expression of both UPR and autophagy-associated key regulatory proteins occurs with more latency compared to gene expression [4, 5] and in the present study we were unable to detect any alterations in protein expression at 18 hours after exercise. This limitation biopsy timing was the consequence of leveraging existing biospecimens from the previous study design for which biopsy timing was optimized for the measurement of protein fractional synthesis rates. Nonetheless, we feel that the transcriptional evidence for an age-related attenuation in the adaptive UPR in the present study, in combination with our prior evidence of no age-related differences in protein fractional synthesis rates [24], provides insight on the regulation of muscle protein turnover and quality control in response to exercise. It is also important to consider that dietary intake has strong influence on UPR activation in skeletal muscle [23, 33]. Food intake and macronutrient composition in the present study was carefully controlled for three days prior to and throughout the study in a clinical inpatient setting, thereby decreasing the inherent variability that could potentially obscure age group differences. Finally, the average age of the older cohort in the present study was approximately 15 years older than the group studied by Hentilä and colleagues [4]. Prior evidence supports age-related alterations in stress response pathways within skeletal muscle manifesting after the seventh decade of life [32]. It is therefore likely that a ~15-year difference in age may influence the responsiveness of skeletal muscle to unaccustomed exercise in adults over 70 years of age.

In conclusion, older adults exhibited decreased markers of UPR activation and reduced coordination with autophagy and SC-associated gene transcripts following a single bout of unaccustomed resistance exercise. In contrast, young adults demonstrated strong coordination between UPR genes and key regulatory gene transcripts associated with autophagy and SC differentiation in skeletal muscle post-exercise. Taken together, the present findings suggest a potential age-related impairment in the post-exercise transcriptional response supporting activation of the UPR and coordination with other exercise responsive pathways (i.e., autophagy, SC differentiation) in skeletal muscle that is likely to contribute to sarcopenia and age-related attenuation of adaptive responses to exercise.

## MATERIALS AND METHODS

The experimental design has been previously described [24]. In brief, twelve young (18-35 years) and 12 older (65-85 years) men and women were recruited and provided written informed consent as approved by the Mayo Foundation Institutional Review Board. The study conformed to the principles outlined in the Declaration of Helsinki. All participants completed a screening visit followed by a two-day inpatient study to evaluate acute exercise responsiveness. Participants were included in the study only if they did not participate in routine exercise training more than two days per week to limit the influence of a training effect on UPR responsiveness to a single bout of unaccustomed resistance exercise [4]. Those who met the eligibility reported to the Clinical Research and Trials Unit (CRTU) at Mayo Clinic Hospital, St. Marys Campus following an overnight fast. The screening visit consisted of a physical examination, comprehensive blood tests, dual-energy X-ray absorptiometry (DEXA) to determine whole-body fat and fat free mass (FFM), and physical function tests. Physical function tests consisted of a whole-body peak oxygen uptake (VO_2_ peak) assessment performed on a stationary cycle ergometer and a maximal knee extensor strength assessment, evaluated on two separate occasions, to calculate the one repetition maximum (1RM) using methods previously described [24]. To calculate 1RM, participants performed a warm-up of 10 repetitions on a plate-loaded knee extensor apparatus with a relatively low weight, followed by 2 sets of up to 10 repetitions with 3 min recovery intervals between sets. 1RM was calculated from the average of two sets, such that 1RM = ω·(1+*r*/30), where ω is the weight lifted on the plate-loaded knee extensor apparatus in arbitrary units (AU) and *r* is the number of repetitions per set. Leg strength was also normalized to leg FFM (AU/FFM) using results from the DEXA scan.

Prior to the first in-patient study day, participants were admitted to the CRTU at 1700 hrs and consumed nothing but water after 2200 hrs. The following morning, muscle biopsies were obtained from the right *vastus lateralis* at 0800 hrs. Following the biopsy, participants were given a standardized meal containing 10 kcal/kg of 20% protein, 50% carbohydrate, and 30% fat at 1200 hrs. At 1600 hrs, participants performed seated unilateral leg extension using only the left leg consisting of 8 sets of 10 repetitions at 70% of their 1 RM, determined during an outpatient visit, followed by 3 minutes of rest between each set. A second meal was given at 1800 hrs with caloric content to achieve weight maintenance, after which participants remained fasted until the second set of biopsies on the following day on the exercised leg (i.e. left *vastus lateralis*) at 1000 hrs, corresponding to 18 hrs post-exercise. Prior evidence suggests minimal differences in baseline gene expression between the right and left legs [50], such that exercise-induced changes in gene expression in one leg can be confidently compared to baseline gene expression in the opposite leg.

### mRNA sequencing

Total RNA was isolated and RNA libraries were prepared as previously described [24]. In brief, total RNA was isolated from the biopsy collected before exercise and 18 hours post-exercise. Sequencing libraries were prepared with TruSeq RNA Sample Prep Kit v2. Libraries were sequenced on a HiSeq 2000 sequencer using TruSeq SBS sequencing kit version 3 and HCS version 2.0.12.0 software. The RNA-Seq data was analyzed using MAP-RSeq v.1.2.1, the Mayo Bioinformatics Core pipeline. MAP-RSeq consists of alignment with TopHat 2.0.6 against the hg19 genome build and gene counts with the HTSeq software using gene annotation files obtained from Illumina (http://cufflinks.cbcb.umd.edu/). A subset of 75 genes associated with the UPR pathway was derived from Reactome (http://www.reactome.org/) (RRID:nif-0000-03390) [51]. Gene transcripts associated with autophagy, the UPR, or mitochondrial biogenesis that were statistically up- or downregulated with different phenotypes (young vs older adults and baseline vs exercise) were subjected to pathway analysis using WEBGESTALT software. GSEA for UPR pathway activation was performed using Broad’s GSEA software. All enrichment p values were FDR corrected (using Benjamini-Hochberg procedure). Genes with a FDR-corrected p value of ≤ 0.05 and an absolute fold change of ≥ 1.20 (where 0.00 signifies no change) were considered for further analysis.

### Quantitative RT-PCR for RNA-Seq validation

Quantitative (q)RT-PCR was performed on transcripts of interest that were identified in the RNA-Seq dataset using procedures we have been previously described [24]. Gene-specific primers are shown in Supplementary Table 1. We verified 9 mRNAs associated with the three primary transducers of the UPR (Supplementary Figure 1). Total RNA was isolated using the RNEasy fibrous tissue kit according to the manufacturer’s instructions. RNA quantity and purity were assessed by spectrophotometric analysis (Nanodrop) in which both the ratios of absorbance at 260 nm to that at 230 nm (A260/230) and the absorbance at 260 nm to that at 280 nm (A260/280) were >1.8. cDNA synthesis was performed using SuperScript III First-Strand Synthesis System for RT-PCR cDNA Synthesis Kit (Invitrogen, according to the manufacturer’s protocol. The cDNA-equivalent of 5 ng RNA was used for amplification in 384-well microtiter plates in a QuantStudio 7 cycler (Applied Biosystems, CA, USA) using SYBR green assays. Cycle threshold (CT) values for individual reactions were normalized against β2 microglobulin expression. All cDNA samples were amplified in duplicate. Relative expression was calculated using the ∆CT method. Data are presented as fold change compared with control, obtained using the ∆∆CT method.

### Western blot analysis

Frozen muscle tissue was pulverized in liquid nitrogen and homogenized at 4°C in modified RIPA buffer (150 mM NaCl, 50 mM Tris-HCl pH 8.0, 1.0 mM EDTA, 1.0% (v/v) Nonidet P40) and phosphatase and protease inhibitors (Halt Protease and Phosphatase Inhibitor Cocktail, Thermo Scientific, Catalog #78446). Homogenates were incubated on ice for 5 min, followed by centrifugation at 10,000*g* to remove insolubilized fragments, and protein was quantitated (Pierce BCA Protein Assay Kit, Thermo Scientific, Catalog #23225). Samples were prepared for western blot in lithium dodecyl sulfate sample buffer (NuPAGE LDS Sample Buffer, Invitrogen, Catalog #NP0007) with 20mM DTT. Samples were heated at 90°C for 10 min, and 22.5 μg protein was added to each well of precast gels (NuPAGE Novex Bis-Tris Mini Gels, Invitrogen). Proteins were separated by electrophoresis and blotted on Immuno-Blot PVDF (Bio-Rad, Catalog #1620177). Membranes were then blocked with Odyssey blocking buffer (LI-COR, Catalog #927-50000) for two hours before incubating overnight with primary antibodies against CHOP (Cell Signaling, Catalog #2895), GRP78 (Santa Cruz Biotechnology, Catalog #sc-376768), p53 (Cell Signaling, Catalog #2524), PDI (Cell Signaling, Catalog #3501) or vinculin protein (EMD Millipore, Catalog #CP74). Proteins were detected using either chemiluminescent or fluorescent methods using anti-mouse or rabbit secondary antibodies. Signal intensity was determined using LI-COR 5.2.5 imaging software.

### Statistical methodology

Unpaired t-tests were used to compare subject characteristics between age groups. For variables measured at baseline and post-exercise, young and older groups were compared using two-way ANOVA (age x time). When variables were not normally distributed, the Wilcoxon signed rank test was used for comparisons pre-post-exercise for both age groups. Pre- and post-exercise values were compared within age groups using paired t-tests and across age-groups using ANOVA with Tukey’s procedure to maintain 5% type 1 error rate for post-hoc testing. Exercise effect on mRNA was evaluated from pre- to post-exercise change using two-way ANOVA (age x time). Statistical analysis was performed using JMP 10 Software (SAS Institute, Cart, NC) and PRISM v7.0e (GraphPad Software Inc, La Jolla, CA).

## Supplementary Material

Supplementary Figures

Supplementary Table 1
