# Supplementary material for: Attenuated activation of the unfolded protein response following exercise in skeletal muscle of older adults
Source: Aging (Albany NY). 2019 Sep 14;11(18):7587–604. doi: 10.18632/aging.102273 (PMC6781982; doi:10.18632/aging.102273)
Supplement: Supplementary Figures [file aging-11-102273-s002.pdf]

SUPPLEMENTARY FIGURES

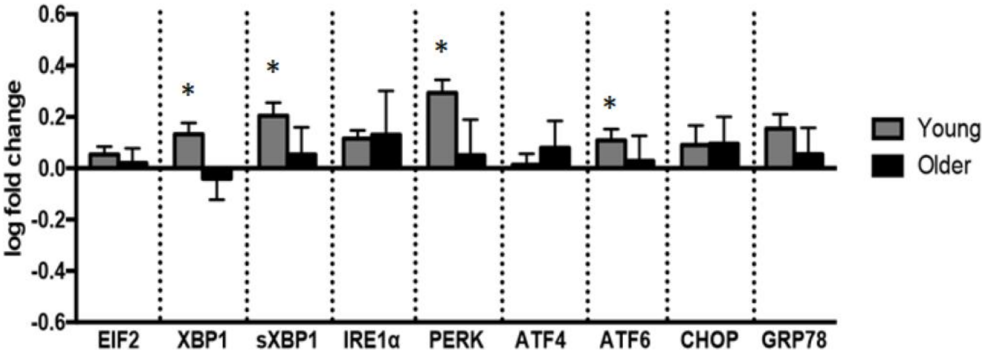

Supplementary Figure 1. qRT-PCR validation of RNA-Seq. We verified nine mRNAs associated with the three primary transducers of the UPR. qPCR, quantitative PCR. *Significant ($p \leq 0.05$) log fold change from baseline within group. Data are means ± SEM.

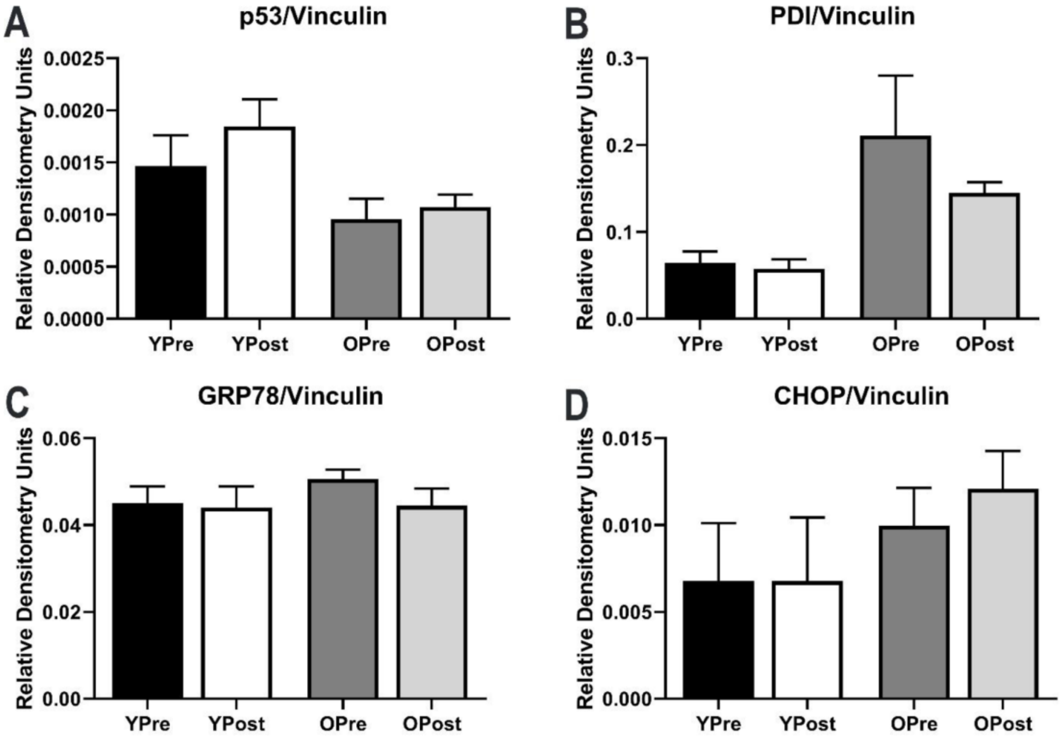

Supplementary Figure 2. Protein expression of p53 (**A**), PDI (**B**), UPR chaperone GRP78 (**C**), and PERK arm-associated terminal protein CHOP (**D**) in young (Y) and older (O) adults at baseline (Pre) and 18h post-exercise (Post). P53 and PDI protein expression was completed for six young and six older adults. CHOP and GRP78 protein expression were completed for three young and three older adults.

Older

	GRP78	PERK	EIF2a	ATF4	CHOP	GADD34	ATF3	p21	p53	SESN1	CASP3	GADD45A	GADD45B	GADD45G	BECN1	BCL2	ATG7	CYCS
GRP78		0.42	0.64*	0.31	-0.10	0.37	0.70*	0.68*	0.64*	-0.18	0.68*	0.53	0.51	0.50	-0.38	-0.62*	-0.19	0.48
PERK	0.72*		0.65*	0.19	0.31	0.36	0.58*	0.54	0.57	-0.13	0.49	-0.07	0.19	-0.10	-0.55	-0.38	-0.12	0.55
EIF2a	0.80*	0.52		0.47	0.12	0.38	0.70*	0.57	0.76*	-0.27	0.53	0.03	0.52	0.11	-0.54	-0.70*	-0.27	0.88*
ATF4	0.41	0.14	0.26		0.33	0.64*	0.39	0.09	0.39	0.17	0.42	-0.12	0.57	-0.09	-0.24	-0.29	-0.08	0.65*
CHOP	0.78*	0.50	0.60*	0.28		0.59*	-0.09	-0.09	-0.07	0.32	0.35	-0.40	0.31	-0.67*	0.19	-0.12	-0.24	0.30
GADD34	0.84*	0.60*	0.65*	0.71*	0.65*		0.33	0.15	0.38	0.17	0.60*	-0.15	0.60*	-0.18	0.00	-0.25	-0.15	0.65*
ATF3	0.75*	0.70*	0.81*	0.43	0.51	0.76*		0.84*	0.94*	-0.63*	0.72*	0.45	0.45	0.53	-0.48	-0.77*	-0.16	0.64*
p21	0.73*	0.74*	0.76*	0.09	0.44	0.48	0.85*		0.75*	-0.66*	0.64*	0.48	0.46	0.55	-0.61*	-0.80*	-0.18	0.47
p53	0.81*	0.84*	0.59*	0.23	0.51	0.55	0.60*	0.73*		-0.57	0.57	0.26	0.50	0.43	-0.51	-0.74*	-0.26	0.70*
SESN1	-0.71*	-0.56	-0.67*	-0.14	-0.31	-0.47	-0.62*	-0.68*	-0.76*		-0.33	-0.58*	-0.11	-0.56	0.13	0.49	0.29	-0.21
CASP3	0.81*	0.60*	0.87*	0.42	0.61*	0.71*	0.89*	0.79*	0.70*	-0.69*		0.38	0.58*	0.16	-0.11	-0.71*	-0.07	0.64*
GADD45A	0.93*	0.66*	0.83*	0.43	0.76*	0.80*	0.75*	0.73*	0.80*	-0.67*	0.85*		-0.05	0.76*	0.16	-0.18	-0.27	-0.13
GADD45B	0.87*	0.43	0.73*	0.57	0.63*	0.87*	0.70*	0.52	0.48	-0.57	0.67*	0.76*		-0.10	-0.42	-0.67*	-0.34	0.64*
GADD45G	-0.79*	-0.64*	-0.51	-0.27	-0.73*	-0.59*	-0.59*	-0.57	-0.71*	0.48	-0.73*	-0.66*	-0.59*		-0.14	-0.28	0.10	0.00
BECN1	-0.73*	-0.52	-0.52	-0.69*	-0.62*	-0.76*	-0.66*	-0.41	-0.59*	0.43	-0.73*	-0.65*	-0.73*	0.80*		0.48	0.00	-0.40
BCL2	-0.46	-0.61*	-0.66*	0.32	-0.31	-0.24	-0.70*	-0.79*	-0.45	0.56	-0.64*	-0.44	-0.26	0.43	0.15		0.08	-0.69*
ATG7	-0.29	-0.41	-0.07	0.01	-0.35	-0.01	-0.12	-0.39	-0.51	0.22	-0.09	-0.37	-0.05	0.18	0.14	0.00		-0.09
CYCS	0.34	0.31	0.76*	-0.02	0.27	0.23	0.73*	0.71*	0.22	-0.42	0.69*	0.41	0.28	-0.21	-0.19	-0.79*	0.02	

Young

Supplementary Figure 3. Pearson R correlation values between gene transcripts associated with the PERK arm of the UPR and p53/p21 axis post-exercise in young (lower left) compared to older (upper right) adults. The correlation vales demonstrate relationships displayed in Figure 4C between the fold-change from baseline to 18h post-exercise for the individual gene transcripts associated with the UPR pathway in young and older adults. *Significant ($p \leq 0.05$) correlation.
