# Supplementary material for: Attenuated activation of the unfolded protein response following exercise in skeletal muscle of older adults
Source: Aging (Albany NY). 2019 Sep 14;11(18):7587–604. doi: 10.18632/aging.102273 (PMC6781982; doi:10.18632/aging.102273)
Supplement: Supplementary Table 1 [file aging-11-102273-s001.pdf]

SUPPLEMENTARY TABLE

Supplementary Table 1. Primer sequences for quantitative RT-PCR.

Gene	Forward (5′ – 3′)	Reverse (5′ – 3′)
Grp78	CGACTCGAATTCCAAAGATTCA	CCTGGACAGCAGCACCATAC
CHOP	AGCTGGAAGCCTGGTATGA	TTTCCAGGAGGTGAAACATAG
ATF4	CCCTCCAACAACAGCAAGGAGGA	ACCCAACAGGGCATCCAAGTCA
ATF6	CCCGTATTCTTCAGGGTGCTCTGG	TAGCTCACTCCCTGAGTTCCTGCT
PERK	GTTGTCGCCAATGGGATAGT	CGAGGTCCGACAGCTCTAAC
IRE1	GCAAGCTGACGCCCACTCTGTA	AAAGGAAGTGTGCTGCCGCG
XBP1	AGGAGAAGGCGCTGAGGAGGAAACT	ACCACTTGCTGTTCCAGCTCACTCA
Spliced XBP1	TGCTGAGTCCGCAGCAGGTG	GCTGGCAGGCTCTGGGGAAG
eIF2α	GGCCTTTCTGTCCTCAGTCAAGCT	ATCTGTGACCACTTTGGGCTCCAT
B2 Microglobulin	ACTTGTCTTTCAGCAAGGACTG	TTCACACGGCAGGCATACT
